# Exciton Recombination, Energy-, and Charge Transfer in Single- and Multilayer Quantum-Dot Films on Silver Plasmonic Resonators

**DOI:** 10.1038/srep26204

**Published:** 2016-05-17

**Authors:** Taeho Shin, Kyung-Sang Cho, Dong-Jin Yun, Jinwoo Kim, Xiang-Shu Li, Eui-Seong Moon, Chan-Wook Baik, Sun Il Kim, Miyoung Kim, Jun Hee Choi, Gyeong-Su Park, Jai-Kwang Shin, Sungwoo Hwang, Tae-Sung Jung

**Affiliations:** 1 Analytical Science Group, Samsung Advanced Institute of Technology, Suwon 443-803, Republic of Korea; 2 Department of Chemistry, Chonbuk National University, Jeonju 561–756, Republic of Korea; 3 Device & System Research Center, Samsung Advanced Institute of Technology, Suwon 443-803, Republic of Korea; 4 Department of Materials Science and Engineering, Frederick Seitz Materials Research Laboratory, University of Illinois at Urbana-Champaign, Urbana, IL 61801, USA; 5 Department of Materials Science and Engineering, Seoul National University, Seoul 151–744, Republic of Korea

## Abstract

We examine exciton recombination, energy-, and charge transfer in multilayer CdS/ZnS quantum dots (QDs) on silver plasmonic resonators using photoluminescence (PL) and excitation spectroscopy along with kinetic modeling and simulations. The exciton dynamics including all the processes are strongly affected by the separation distance between QDs and silver resonators, excitation wavelength, and QD film thickness. For a direct contact or very small distance, interfacial charge transfer and tunneling dominate over intrinsic radiative recombination and exciton energy transfer to surface plasmons (SPs), resulting in PL suppression. With increasing distance, however, tunneling diminishes dramatically, while long-range exciton-SP coupling takes place much faster (>6.5 ns) than intrinsic recombination (~200 ns) causing considerable PL enhancement. The exciton-SP coupling strength shows a strong dependence on excitation wavelengths, suggesting the state-specific dynamics of excitons and the down-conversion of surface plasmons involved. The overlayers as well as the bottom monolayer of QD multilayers exhibit significant PL enhancement mainly through long-range exciton-SP coupling. The overall emission behaviors from single- and multilayer QD films on silver resonators are described quantitatively by a photophysical kinetic model and simulations. The present experimental and simulation results provide important and useful design rules for QD-based light harvesting applications using the exciton-surface plasmon coupling.

Semiconductor quantum dots (QDs) have attracted great attention in diverse applications ranging from optoelectronic devices such as single photon sources and light emitting diodes (LED) to biological imaging because of their excellent optical properties and ease of chemical synthesis^1^,^2^,^3^. Whether they are pumped by photoexcitation or carrier injection, electron-hole pairs (excitons) are generated and subsequently recombine to emit light through radiative relaxation or to release heat through non-radiative relaxation. Unfortunately, the latter is significant against the former along with ultrafast interfacial exciton dissociation in most applications and imposes severe limitations to high device performances. Many endeavors have been made to enhance radiative recombination or spontaneous emission mostly using chemical methods.

Recently, active manipulation over exciton relaxation kinetics using surface plasmons has been attempted in various optical emitters leading to significant increases in light emission^4^,^5^,^6^,^7^,^8^,^9^,^10^,^11^,^12^,^13^,^14^,^15^,^16^,^17^,^18^,^19^,^20^,^21^. Surface plasmons (SPs) are collective electron oscillations, as one of elementary excitations in metals. They are responsible for surface-enhanced Raman scattering and strong light absorption by molecules on metals by increasing the optical excitation field at the surface greatly^22^,^23^,^24^. On the other hand, the local density of states associated with surface plasmons influences photoexcited molecules by altering their relaxation pathways and kinetics significantly. For example, the apparent emission rate can be greatly enhanced compared to the intrinsic emission rate when surface plasmonic states match with the exciton states of molecules. This is the Purcell effect by surface plasmons.

In this paper, we examine exciton recombination, energy-, and charge transfer in single-and multilayer CdS/ZnS quantum dots (QDs) on silver plasmonic resonators using photoluminescence (PL) and excitation spectroscopy along with photophysical kinetic modeling and simulations. The PL enhancement of QDs by surface plasmons has been reported mostly using simple structures of sub- or full monolayers on metallic films and the scope aimed mainly at revealing the distance dependence and mechanism of exciton-SP coupling. Here, we employ multilayers of QDs on silver resonators in which the exciton dynamics are rich and more complicated and accordingly the overall emission behaviors are significantly altered compared to the sub- or full monolayer structure. Despite the complexity, since the structure is more practical and versatile building blocks for light harvesting applications, the understanding of the dynamics is important. We present experimental and simulation results of the emission dynamics obtained using spectroscopy, kinetic modeling and simulations, elucidating the effects of not only the separation distance but also excitation wavelength, excitation power, and QD film thickness.

## Result and Discussion

### Exciton-SP coupling in QD monolayers on Ag resonators

**Figure 1:**
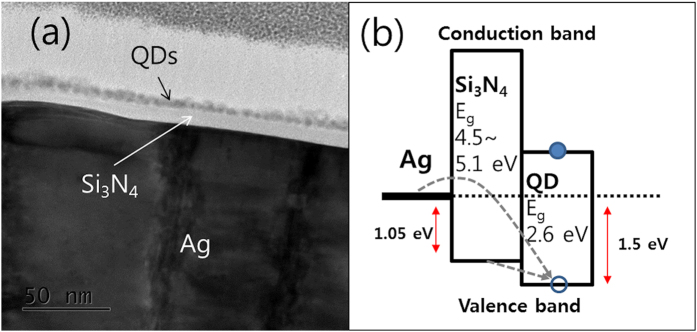
(**a**) TEM image representing a QD monolayer, 20-nm thick Si_3_N_4_ and 300-nm thick Ag films. (**b**) Energy level diagram of QDs, Si_3_N_4_ and Ag films.

A vertical cross-section of the samples is shown by a transmission electron microscopy (TEM) image in Fig. 1(a) 300-nm thick Ag films were prepared on sapphire substrates and insulating silicon nitride (Si_3_N_4_) films with variable thickness (0–100 nm) were deposited on the Ag films. Single- and multilayer QD films (*N* ~ 1, 3, 5, 10, and 30) were spin-coated on the Si_3_N_4_ films. Figure 1(b) depicts electronic energy levels of QDs, Si_3_N_4_, and silver films obtained from X-ray photoemission spectroscopy and UV-visible absorbance measurements. It indicates that excitons in QDs can be dissociated through hole transfer from photoexcited QDs to the insulating Si_3_N_4_ and metallic Ag films.

**Figure 2:**
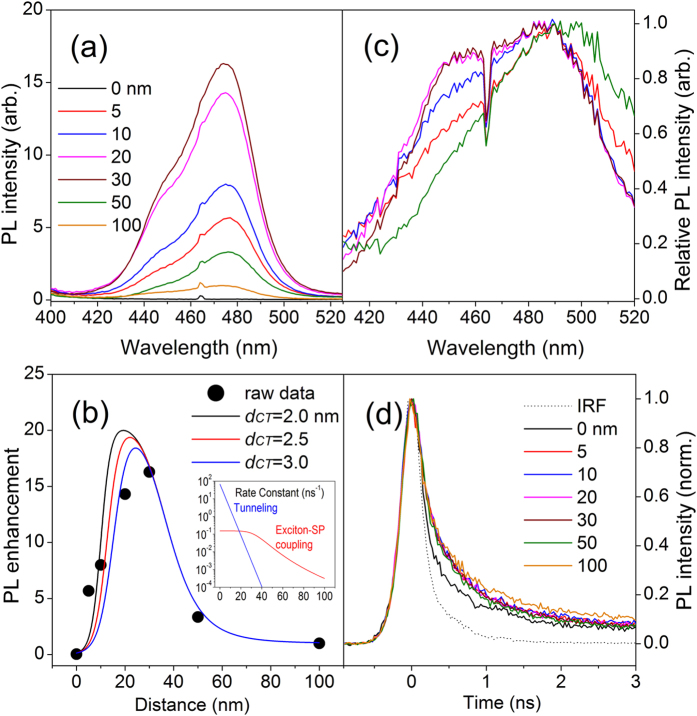
(**a**) Steady-state PL spectra of QD monolayers on the resonator as a function of Si_3_N_4_ thickness. The intensities are divided by the maximum intensity of QDs/100-nm thick Si_3_N_4_/Ag. (**b**) Variation of PL intensities at peak wavelengths with increasing thickness (solid circles) and fitting results (three curves) using a kinetic model (see the details in the modeling and simulation section). The inset shows the distance dependence of the rate constants of charge transfer (blue, *d*_*CT*_ = 3.0 nm) and exciton-SP coupling (red). (**c**) The PL spectra in (**a**) are divided by that of QDs/100-nm thick Si_3_N_4_/Ag for all the PL emission wavelengths and then normalized by their maximum values. (**d**) Time-resolved PL curves. Semi-log plots of the curves are found in Figure S1 in Supplementary Information. The excitation wavelength (of a picosecond laser) was 379 nm. The same color indicates the same sample in (**a,c,d**).

Figure 2(a) presents the variation of the PL spectra from QD monolayers as the insulating Si_3_N_4_ film thickness (*d*) changes from 0 to 100 nm. All of the spectra show maximum intensities near 475 nm, but their intensities are substantially different. When quantum dots are placed directly on the silver film (*d* = 0), PL is strongly quenched and gives negligible intensity. With increasing thickness, PL spectrum continues to grow yielding the maximum intensity at *d* = 30 nm and then diminishes. Figure 2(b) shows the intensity variation at peak wavelengths (475 nm) as a function of Si_3_N_4_ thickness. For 5 nm ≤ *d* ≤ 50nm, spontaneous emission from QDs is enhanced by the silver film. The PL intensity at *d* = 30 nm is ~15 times larger compared to that at *d* = 100 nm. The PL intensity stays at a constant value for *d* ≥ 100 nm.

Based on the observations in Fig. 2(a,b) that PL is almost quenched at *d* = 0 nm and shows a maximum value at *d* = 30 nm, it is inferred that the presence of silver metal opens up two additional competing relaxation channels to photoexcited QDs. One is exciton dissociation via charge transfer and the other one is energy transfer from QDs to the metal via coupling between excitons and surface plasmon modes. The proximity of quantum dots to the silver film influences their relative rates. When QDs are in contact with the silver film, holes transfer from photoexcited QDs to the metal, leading to interfacial exciton dissociation and subsequently causing PL quenching as shown in Fig. 1(b). The insulating Si_3_N_4_ film (*d* > 0) cannot inhibit charge transfer completely due to electron tunneling. Electrons tunnel across the insulating Si_3_N_4_ layer giving rise to exciton dissociation and partial PL quenching for small thicknesses. However, since electron tunneling decays exponentially with the barrier thickness, PL quenching should become weak and the PL intensity grows simultaneously. Even at 5 nm the intensity already surpasses that of QD monolayers on 100-nm thick Si_3_N_4_ films. Interfacial charge transfer from QDs to the Si_3_N_4_ film is also energetically possible in Fig. 1(b), but is not as favorable as for the silver metal because of local charging effects. Therefore, PL quenching is not significant as for the QDs-silver contact.

Coupling between excitons and surface plasmons accounts for the PL enhancement of the QD monolayers. In the absence of the silver metal, QDs rely only on intrinsic radiative recombination for spontaneous light emission against non-radiative recombination. However, when QDs are in the proximity of silver metal, then excitons view it as another channel for relaxation. Instead of relaxing via non-radiative recombination, they can couple with surface plasmon modes and generate surface plasmons. The generated surface plasmons relax to emit light or release heat into the lattice. If the former is dominant, then there is gain in PL intensities. Defective structures and edges of metals play an important role in light emission from surface plasmons^5^,^8^. Even though silver films were prepared using an e-beam evaporation method, they show a high density of nanometer scale surface corrugations which serve as scattering centers of surface plasmons (Figure S2 in Supplementary Information). The energy of surface plasmons is scattered off the defective corrugations into the far-field and enhance light emission of quantum dots.

From the distance dependence of the PL intensity, we deduce the distance dependence of exciton-SP coupling. If coupling is considered as energy transfer via dipole-dipole interactions as for Förster resonance energy transfer (FRET), the coupling efficiency would decay following the same ~*d*^−6^ dependence. The ~*d*^−6^ behaviors were also reported between QDs and metal nanoparticles (MNPs) by Chan *et al*.^11^. Figure 2(b) shows fitting curves assuming the exponential decay of tunneling and the *d*^−6^-dependence of exciton-SP coupling. The exciton-SP coupling time is ~6.5 ns for *d* = 0 nm and the characteristic coupling distance is ~36 nm. The estimated distance is larger compared to typical distances for energy transfer between molecular dyes, but it is comparable to the distance for energy transfer between QDs and MNPs^11^. The SPs penetration depth may also account for the large distance as another coupling mechanism^5^. The tunneling distance is estimated as ~1.5–3.0 nm assuming the charge transfer time of ~15 ps for *d* = 0 nm. More details are described later in the modeling and simulation section in Methods.

Assuming exciton-SP coupling as FRET, the enhanced PL spectra in Fig. 2(a) result from the spectral overlap between PL spectrum of QDs (without coupling with SP) and surface plasmon modes of the silver film. If they are overlapped more at specific energy levels (or wavelengths), the larger PL enhancement is expected. The wavelength dependence of surface plasmon modes at the interface of Si_3_N_4_/Ag may be obtained by dividing the enhanced PL spectra with that of the 100-nm thick sample in which exciton-SP coupling is negligible^5^. This is shown in Fig. 2(c), revealing surface plasmon modes over the PL spectral region. Using dielectric function ε(ω) of silver and that (ε = 6.5–7.2) of Si_3_N_4,_ the surface plasmon energy is estimated as 470–490 nm at the Si_3_N_4_/Ag interface^25^,^26^, which explains the emission wavelength dependence of the enhancement in Fig. 2(c). In fact, the effective dielectric constant may depend on the thickness of Si_3_N_4_ and affect the peak wavelength as a function of the Si_3_N_4_ thickness. However, the dramatic shift of the resonance peak wavelength was not observed over the thickness range employed. The local dielectric constant at the very interface between Si_3_N_4_ and Ag may not vary considerably and so does not change the resonance peak wavelength significantly.

Time-resolved PL decay curves are presented in Fig. 2(d). The observed decay times are approximately ~3 ns. They are not dramatically changed with the film thickness unlike the steady-state PL spectra in Fig. 2(a). The deconvolution using the instrument response function (IRF, dotted line in Fig. 2(d)) and subsequent fitting with multiple exponential functions yield average time constants. More details are described in the curve fitting section in Methods. For *d* = 0 nm, the time constant is estimated shorter than the time resolution (~50 ps), indicating ultrafast electron transfer. The longest time is 3.5 ns for *d* = 100 nm and the shortest one 2.5 ns for *d* = 30 nm, but the difference seems insignificant. This is attributed to the low intrinsic quantum yield (or intrinsic short PL decay time) of the QDs on Si_3_N_4_ films. In general, it is expected that the decay times are shortened significantly for a larger PL enhancement because of the acceleration of the spontaneous emission rate due to exciton-SP coupling^9^,^10^,^15^,^17^. However, when the quantum yield of QDs is low or the non-radiative recombination rate is much faster than the radiative recombination and the exciton-SP coupling rate, shortening of the PL decay times is insignificant even for large PL enhancements. This is verified by the intrinsic fast decay of QDs used in this work and the following kinetic analysis. The intrinsic PL decay time for *d* = 100 nm is only 3.5 ns. This is much shorter compared to typical decay times (~100 ns) for QDs with the high quantum yield^9^.

Without exciton-SP coupling, for example, for *d* = 100 nm, the observed decay time is given by 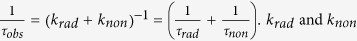 represent the rate constants for radiative- and non-radiative recombination, respectively. *τ*_*rad*_ and *τ*_*non*_ are the time constants for radiative and non-radiative relaxation, respectively. As we discussed, interfacial charge transfer between QDs and the insulating Si_3_N_4_ film can occur and thus must also be considered. Instead of adding the non-radiative term, the rate- and time constants for the non-radiative process are tentatively defined to include both the intrinsic non-radiative recombination and charge transfer terms. The terms will be separated later in multilayer QD measurements. The quantum yield of QDs on the Si_3_N_4_ film is defined as (*k*_*rad*_)/(*k*_*rad*_ + *k*_*non*_). In the presence of exciton-SP coupling, the PL decay time is modified as (1)/(*τ*_*obs*_) = (*k*_*rad*_ + *k*_*non*_ + *k*_*sp*_)^−1^ = ((1)/(*τ*_*rad*_) + (1)/(*τ*_*non*_) + (1)/(*τ*_*SP*_)). Here, *k*_*SP*_ and *τ*_*SP*_ are the rate constant and time constant for the coupling, respectively. The quantum yield becomes (*k*_*rad*_ + *k*_*SP*_)/(*k*_*rad*_ + *k*_*non*_ + *k*_*SP*_) assuming that all of the generated SPs relax to emit light. Therefore, the PL enhancement ratio is given by ((*k*_*rad*_ + *k*_*SP*_)/(*k*_*rad*_ + *k*_*non*_ + *k*_*SP*_))/((*k*_*rad*_)/(*k*_*rad*_ + *k*_*non*_)). When *k*_*rad*_ ≪ *k*_*non*_ and *k*_*rad*_ ≪ *k*_*SP*_, it is approximated to ~(*k*_*SP*_)/(*k*_*rad*_). From simple calculations using the constants and data from Fig. 2(b), it is found that *τ*_*rad*_ ≈ 200 ns,*τ*_*non*_ ≈ 3.5 ns, and *τ*_*SP*_ ≈ 8.8 ns for *d* = 30 nm In this case, the quantum yield increases from 1.7% to 28% owing to the silver resonator. F or *d* = 50 nm, *τ*_*SP*_ ≈ 56.5 ns. It is noticeable that the timescale for *τ*_*SP*_ coincides with the typical energy transfer time in FRET in agreement with previous results^11^,^27^. In contrast to the high quantum yield (40–50%) of original quantum dots in solutions, the low quantum yield of 1.7% was obtained in the structure without silver resonators. This originates from degradation of quantum dots during or after device fabrication. This enhancement scheme can be extended in other diverse QD devices so that a lowered quantum yield due to inevitable degradation can be compensated and enhanced by employing surface plasmon structures. Using the intensity ratio in Fig. 2(a) and the time constants obtained above, the interfacial electron transfer time (*d* = 0), *τ*_*CT*_ is estimated as ~15 ps. Kinetics and the associated parameters are described in detail in the modeling and simulation section.

### Influences of excitation wavelength and power on exciton-SP coupling

**Figure 3:**
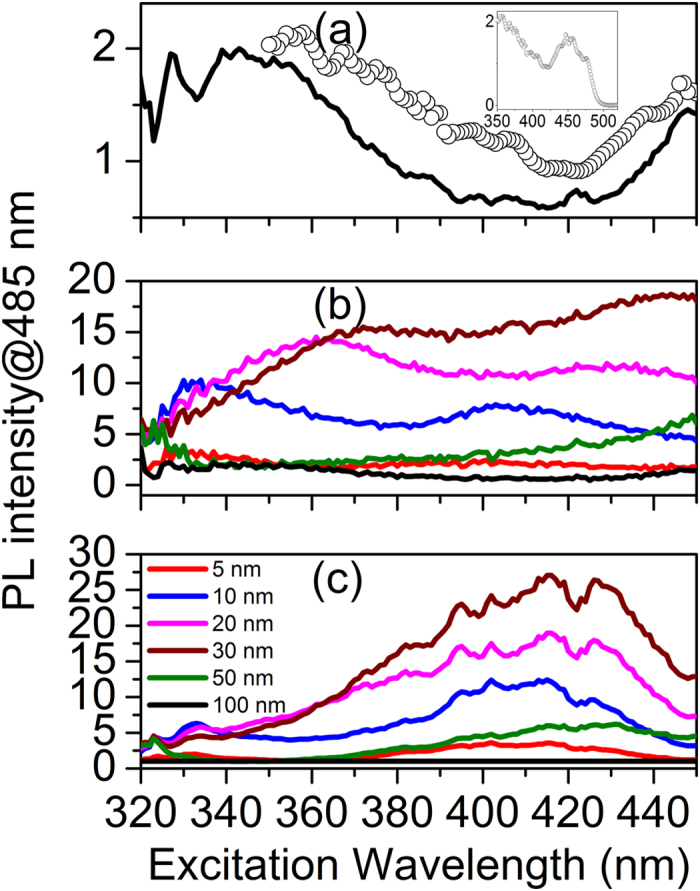
(**a**) Excitation spectrum of a QD monolayer (black line) and differential absorption spectrum of a QD solution (open circles in an arbitrary scale). The black line indicates the variation of PL intensity at 485 nm from the QD monolayer on 100-nm thick Si_3_N_4_/Ag as a function of excitation wavelength. The open circles form a differential absorption spectrum (dA/dλ), which is a measure of density of states (DOS) of QDs as a function of wavelength. The inset is the differential absorption spectrum with a wider wavelength range. (**b**) Excitation spectra of the samples (QD monolayers on 5-nm, 10-nm, 20-nm, 30-nm, 50-nm and 100-nm thick Si_3_N_4_/Ag). All the intensities are re-scaled simultaneously for comparison such that the intensity of QDs on 100-nm thick Si_3_N_4_/Ag is adjusted to 1 for the excitation at 379 nm. (**c**) Re-plot of the PL intensities in (**b**) by normalizing with the intensity from the QD monolayer on 100-nm thick Si_3_N_4_/Ag at all the excitation wavelengths. The same color indicates the same sample in (**a–c**).

To examine the effects of excitation variables on the PL intensity, we varied the excitation wavelength first using a xenon lamp while measuring the PL intensity at 485 nm near the peak wavelength. Figure 3(a) represents the excitation spectrum of the QD monolayer on 100-nm thick Si_3_N_4_/Ag, which reflects mainly absorption features of QDs due to the negligible PL enhancement. Indeed, the differential absorption spectrum (open circles) explains the excitation spectrum well. Figure 3(b) shows the excitation spectra of all the samples with different thickness of the Si_3_N_4_ film. All the intensities are adjusted simultaneously for comparison such that the intensity of QDs on 100-nm thick Si_3_N_4_/Ag is equal to 1 for excitation at 379 nm, so the intensity in y-axis represents the PL enhancement ratio with respect to the specific value. They are expected to exhibit the wavelength dependence that should originate from the absorption features of QDs in Fig. 3(a), but the excitation spectra in Fig. 3(b) show big discrepancies compared to the spectrum of Fig. 3(a). The high PL intensity near 340 nm to 370 nm in Fig. 3(b) is evidently attributed to strong light absorption of QDs as shown in Fig. 3(a). However, the maximum position is observed to red-shift with increasing Si_3_N_4_ film thickness which is not explained by the absorption feature. On the other hand, the intensity around 400 nm to 430 nm (Fig. 3(b)) stays high or even increases despite low light absorption of QDs. These behaviors suggest that the excitation energy may influence the exciton-SP coupling strength (of a single exciton generated).

The wavelength-dependent absorption feature can be removed from the excitation spectra to extract out only the wavelength dependence of exciton-SP coupling, by dividing the excitation spectra by that of the QD monolayer on 100-nm thick Si_3_N_4_/Ag. The processed excitation spectra are shown in Fig. 3(c). They can be considered as the exciton-SP coupling strength as a function of the excitation wavelength, revealing how efficiently a single exciton generated at a specific wavelength is coupled to SP modes and eventually emit a photon compared to intrinsic radiative recombination. Unlike the excitation spectra shown in Fig. 3(b), the processed spectra show common features unambiguously. They continue to grow until ~420 nm as the wavelength increases and then diminish. It appears that the wavelength for the maximum enhancement (for a single exciton generated) shifts towards longer one with increasing Si_3_N_4_ film thickness. For example, the maximum values are observed around ~410 nm for 5-nm thick Si_3_N_4_ and ~430 nm for 50-nm thick Si_3_N_4_, respectively.

For a fixed excitation wavelength (379 nm), the emission wavelength dependence in Fig. 2(c) reveals mainly the coupling strength of excitons near the band edge after intraband relaxation, whereas the excitation wavelength dependence in Fig. 3(c) is associated with exciton states and the dynamics during intraband relaxation for a given emission wavelength (485 nm). To address the excitation wavelength dependence, the state-specific dynamics of electrons should be considered.

In general, when semiconductors are excited by photons whose energy is higher than the band-edge transition, then hot electrons (or holes) are generated and then undergo intraband relaxation. Such hot electron-hole pairs may be considered to couple with surface plasmon modes for the light emission. However, this is unlikely because intraband relaxation takes place usually on picosecond time scales which is much faster than the exciton-SP coupling time (as estimated as several nanoseconds or longer here)^28^,^29^.

For QDs, there are discrete electronic energy levels above the band edge and the interval between them is significant. These states are achievable in the course of intraband relaxation or direct resonant excitation. The life times associated with the levels can be comparable to the exciton-SP coupling time^29^. The excitation wavelength dependence may be attributed to upper-state excitons for exciton-SP coupling and subsequent plasmonic down-conversion. We presume that upon photoexciation in the range of 400–420 nm, excitons are generated at the levels and stay with certain life times before complete intraband relaxation. In the presence of the metal resonator, those states are possibly coupled with SP modes competing against internal non-radiative relaxation or interfacial non-radiative relaxation due to the Si_3_N_4_ substrate. Then upper state exciton-SP coupling is followed by plasmonic down-conversion. Without the down conversion, the emission wavelength would be shorter than that of the band-edge emission, but such spectral components are not observed in the range of ~400–420 nm. The down-conversion of the coupled exciton-SP states may also be possible. The down-conversion is not directly evidenced in this study but is strongly implied.

**Figure 4:**
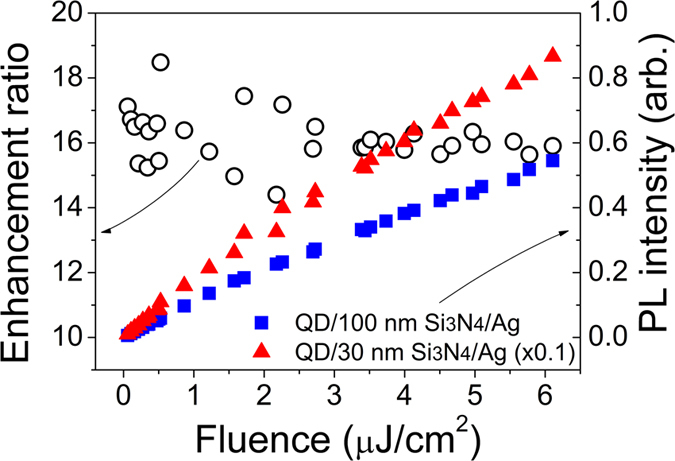
Influence of excitation flunece (~50 ps laser pulses at 405 nm) on the PL intensity at 475 nm. It represents the variations of the PL intensities of QDs on 100-nm thick Si_3_N_4_/Ag (blue squares) and of QDs on 30-nm thick Si_3_N_4_/Ag (red triangles) with increasing fluence, respectively. Their relative intensity ratio defines the enhancement ratio indicated by the open circles.

In contrast to Raman scattering and absorption (at metal interfaces) enhanced by the excitation field due to the incident light^22^,^23^,^24^, the PL enhancement results from energy transfer from QDs to surface plasmon modes but not from such field-induced effects. To check whether the excitation field plays any important roles in enhancing the PL intensity, we investigated the excitation-power-dependent behaviors of the PL intensity as shown in Fig. 4. The excitation wavelength was selected at 405 nm where the PL intensity is significant in as shown in Fig. 3(b). Both of the PL intensities from the QD monolayers on 100-nm thick Si_3_N_4_/Ag (squares) and from that on 30-nm thick Si_3_N_4_/Ag (triangles) increase linearly with increasing fluence. However, their relative ratio stays constant around ~16 regardless of fluence as indicated by open circles. The constant ratio suggests that the stronger excitation field does not enhance exciton-SP coupling more than the weaker field does and that the PL enhancement results from energy transfer from QDs to SP modes rather than from the field-induced effects as in Raman scattering or absorption.

### PL enhancement of QD multilayers on silver resonators

**Figure 5:**
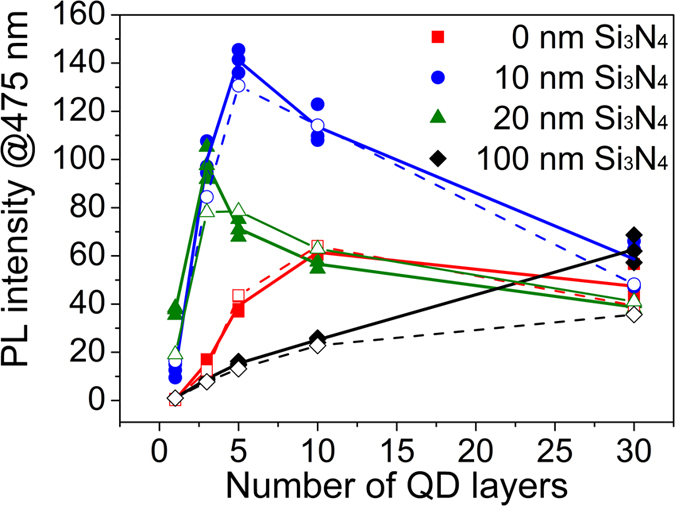
Variations of the PL intensity with increasing QD layers for several thicknesses of the Si_3_N_4_ film. The solid and open shapes (square, circle, triangle, and diamond) indicate experimental data and fitting results, respectively. PL measurements (photoexcitation at 379 nm) at three different probe spots (~3 × 3 mm^2^) were made and those results are represented for showing spatial fluctuations. All the PL intensities (y-axis) are adjusted such that the intensity from the QD monolayer on 100-nm thick Si_3_N_4_/Ag is 1. The fitting scheme is described in detail in the modeling and simulations section.

Although exciton-SP coupling reduces with increasing separation distance, it is still significant at large distance as shown in Fig. 2(a,b). This strongly implies that not only the first monolayer but also upper layers of multilayer QD films can be coupled to surface plasmon modes to increase the total PL intensity greatly. As expected, the total PL intensity increases significantly due to exciton-SP coupling as presented in Fig. 5.

For *d* = 100 nm (without PL enhancement), the PL intensity grows with increasing number of layers. Compared to the intensity from the monolayer, that from five layers increases almost 15 times larger but not simply 5 times. This indicates that the PL intensity of the monolayer is suppressed more at the QD/Si_3_N_4_ interface due to interfacial charge transfer as shown in Fig. 1(b). That is, the external non-radiative recombination due to interfacial charge transfer is dominant for the bottom monolayer compared to intrinsic non-radiative relaxation.

For *d* = 10 nm (with large PL enhancement), the multilayers contribute to the total intensity significantly. The maximum intensity is observed for five QD layers and it is about 10 times larger compared to that from the monolayer. This reveals that exciton coupling with SP modes is not restricted only to the first monolayer but also to the upper layers. In case that excitons are generated in the upper layers, two possible pathways can be considered for the PL enhancement. One is direct long-range coupling to silver SP modes being screened by the lower QD layers plus the 10-nm thick Si_3_N_4_ film. In this case, the excitons view the lower lying QDs as simple dielectric layers as in the same manner as the insulating Si_3_N_4_ film. The other one is multiple energy transfer to the lower lying QDs and subsequent exciton-SP coupling near the Si_3_N_4_ film (or Ag film). Both mechanisms should be possible competing with each other. Since energy transfer takes place between QDs typically on several to tens nanoseconds, which is also comparable to the exciton-SP coupling time as estimated earlier (>~6 ns), the location of excitons matters. If excitons are formed, for example, on the second layer from the bottom, both the pathways would be significant. However, if excitons are formed away from the bottom layer, direct long-range coupling seems dominant because it would take more time for excitons to reach the interface through multiple downward energy transfer among QDs. Moreover, since excitons at the Si_3_N_4_ interface undergo the interfacial non-radiative relaxation, long-range coupling contributes to the total intensity more.

For *d* = 20 nm, the overall behaviors are similar to those for *d* = 10 nm, but the maximum intensity is obtained for three QD layers, which is two-layer thinner compared to the sample with *d* = 10 nm. This is because the thicker Si_3_N_4_ film weakens the coupling strength more significantly already and thus the thinner QD layer is needed for the maximum intensity.

It is interesting that the PL enhancement is observed for the multilayers even in the absence of the Si_3_N_4_ film. PL is suppressed substantially when the QD monolayer is directly on the silver film because of interfacial exciton dissociation as shown in Fig. 2(a,b). The first monolayer undergoes charge transfer dominantly, but direct exciton dissociation through electron tunneling is less likely for the upper layers due to screening by lower lying QD layers. Therefore, the chance for exciton-SP coupling would be higher and cause the PL enhancement. The PL intensity is still significantly lower if it is compared to that from the multilayers on thin Si_3_N_4_ films (*d* = 10 and 20 nm). This is probably due to less efficient exciton-SP coupling through the dielectric medium of QDs. It might be fair to compare the PL intensity from about three QD layers on the silver film to that from the QD monolayer on 10-nm thick Si_3_N_4_/Ag. Indeed, the PL intensity from three QD layers on the silver film is not smaller than that from the QD monolayer on 10-nm thick Si_3_N_4_/Ag, suggesting that the bottom two QD layers play roles effectively similar to the 10-nm thick Si_3_N_4_ for electron tunneling and for exciton-SP coupling.

**Figure 6:**
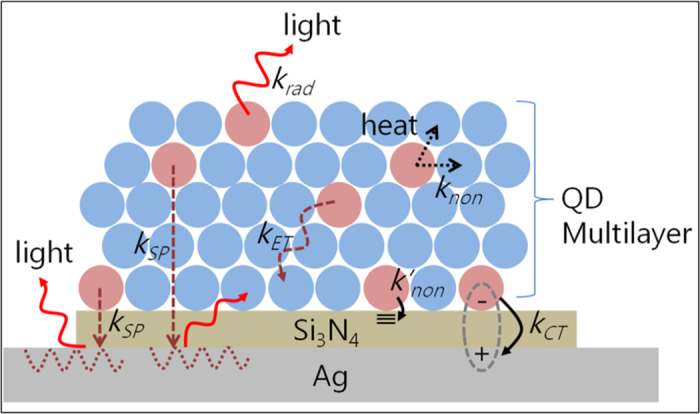
Summary of elementary relaxation pathways of excitons on silver plasmonic resonators. Those include radiative- (*k*_*rad*_) and non-radiative recombination (*k*_*non*_), exciton dissociation by charge transfer to the silver metal (*k*_*CT*_), energy transfer between QDs (*k*_*ET*_), and exciton-SP coupling (*k*_*SP*_). Non-radiative relaxation due to electronic states of Si_3_N_4_ is denoted by *k*′_*non*_.

In the presence of insulating Si_3_N_4_ films (*d* = 10 and 20 nm), the PL intensity grows initially with increasing number of QD layers and then reduces. The behaviors are explained qualitatively in terms of the distance dependence of exciton-SP coupling and the optical skin depth of QD films. If the thickness of QD films is small enough that excitons can couple to SP modes (but long enough that electron tunneling is ignored), then the PL intensity continues to grow with increasing QD film thickness because more QDs and excitons are available for coupling with SP modes. The intensity contributed by intrinsic radiative recombination also increases. However, as the thickness becomes larger and comparable to the optical skin depth, the intensity contributed by exciton-SP coupling becomes weaker because the density of photogenerated excitons decays exponentially along the depth from a maximum value at the top surface and thus the number of excitons is smaller near the interface. On the contrary, intrinsic radiative recombination yields similar or at most slightly higher PL. As a consequence, the total PL intensity would become lower with increasing number of layers as observed. By taking into account all the distance-dependent processes and by varying the associated rate constants, the total PL intensities are fitted numerically as shown in Fig. 5. The fitted results are presented by the open shapes and dashed lines, indicating good agreement with experimental data. All the details for the modeling, numerical simulations, and the rate constants are described in the next section. The elementary relaxation pathways of excitons and associated rate constants are summarized in Fig. 6.

In conclusion, we examined exciton recombination, energy-, and charge transfer between quantum dots and silver plasmonic resonators using photoluminescence (PL) and excitation spectroscopy along with modeling and simulations based on photophysical kinetics. Significant increases in the spontaneous emission of quantum dots were demonstrated by manipulating relaxation pathways and kinetics varying the insulating film thickness, QDs film thickness, and excitation wavelength. Steady-state and time-resolved photoluminescence measurements revealed that energy transfer from photoexcited QDs to surface plasmon resonance states takes place on the order of several nanoseconds (~6 ns), competing with non-radiative recombination (~3.5 ns) and interfacial exciton dissociation (~15 ps) right on the silver resonator. Excitation-dependent measurements showed that the SP coupling efficiency of a single exciton generated is strongly influenced by the photon energy. This is associated with the state-specific dynamics of excitons involving the interface, which allows energy transfer from upper-level excitons with finite life times to surface plasmon modes followed by the down-conversion. The total PL intensity increases greatly using QD multilayer structures due to the enhancement via long-range exciton-SP coupling and intrinsic radiative recombination. Photophysical kinetic modeling and simulations explain the overall emission behaviors of excitons quantitatively well. Our experimental and simulation data will provide important guidelines in achieving the near-unity quantum yield of QDs and in enhancing performances of quantum-dot-based optoelectronic devices as well as of blue optoelectronic devices^30^,^31^,^32^,^33^.

## Methods

### Sample preparation

300-nm thick silver films were prepared on sapphire substrates by electron beam evaporation and Si_3_N_4_ films with variable thicknesses (0 to 100 nm) were grown on the silver films by plasma enhanced chemical vapor deposition (PECVD). The surface morphology of silver films was measured by atomic force microscopy (AFM) as shown in Figure S2 in Supplementary Information. The core/shell CdS/ZnS quantum dot monolayers and multilayers were prepared using a spin coating method. The overall structures were measured by transmission electron microscopy (TEM) as shown in Fig. 1(a) and in Figure S3 in Supplementary Information.

### Spectroscopic measurements

The chemical and electronic structures of QDs, Si_3_N_4_, and silver films were measured using X-ray photoemission spectroscopy (XPS, Quantum 2000) in Figures S4 and S5 in Supplementary Information. The XPS measurements were performed using Alkα source (1486.6 eV) under UHV (<10^−8^ Torr) conditions. The XPS spectra were shifted in x-axis (energy axis) using the known emission peak from C-C bonding (284.8 eV) and rescaled with respect to the Fermi level of the metal (0 eV). The resulting spectra provided the electronic energy levels as presented by Fig. 1(b). Steady-state and time-resolved PL measurements were performed using a spectrometer (FuoTime 300 from PicoQuant). A picosecond laser with a center wavelength of 379 nm or 405 nm was used for photoexcitation of QDs. The laser repetition rate was variable from 196 kHz to 80 MHz and the pulse duration was about 50 ps. A Peltier cooled photomultiflier tube (PMT) was used as a detector. For time-resolved PL, the time-correlated single photon counting (TCSPC) scheme was employed using an electronic module (PicoQuant). The overall instrument response time was about 200 ps and the ultimate time resolution was enhanced up to ~50 ps through the deconvolution procedure. Excitation-wavelength-dependent measurements were also carried out using a xenon arc lamp and the same spectrometer.

### Curve fitting for multiple exponential decay functions

Since two decay components were not enough to fit the PL decay curves in Fig. 2(d), we allowed up to three exponential functions (triple exponential functions). It is not trivial to identify the origin of the three terms, but inhomogeneity of quantum dots used in this study may be responsible for the multiple decay behaviors. The curves are fitted using the linear combination of three exponential functions and the instrument response function as follows.


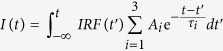


Average decay times (amplitude-weighted average) were obtained as follows.


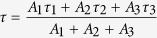


Semi-log plots of the PL decay curves are shown in Figure S1 in Supplementary Information. Temporal behaviors are highly non-linear because of multiple decay components.

### Kinetic modeling and simulations for single- and multilayer QDs on silver resonators

The temporal evolution of the density of excitons at arbitrary locations can be described using photophysical kinetics. At a fixed Si_3_N_4_ thickness (*d*) and a total number of QD layers (*N*), the rate equations are written as follows.


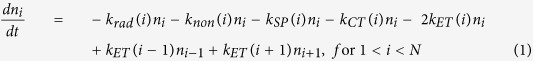



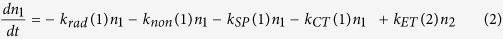



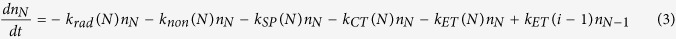



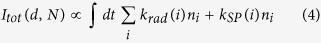


*n*_*i*_ is the density of excitons of the *i*^*th*^ QD layer from the bottom and *N* indicates the top layer. *k*_*rad*_ is the rate constant for radiative recombination, *k*_*non*_ is that for non-radiative recombination, *k*_*SP*_ is that for exciton-SP coupling, *k*_*CT*_ is that for charge transfer to the metal, and *k*_*ET*_ is that for energy transfer between adjacent QDs. Equation (1), (2), and (3) describe how all the competing processes affect the density of excitons temporally. The equation for *n*_*i*_ is coupled with those for *n*_*i*−1_and *n*_*i*+1_ through the energy transfer terms.

The initial condition for *n*(i, t = 0) can be established as an exponential function with the optical skin depth as follows.


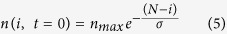



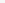
σ is the dimensionless optical skin depth (real skin depth divided by the QD diameter) and would differ depending on the film density. Using the kinetic constants and the initial condition at fixed *N* and *d*, the coupled Equation (1), (2), and (3) can be numerically solved to yield *n*_*i*_(*t*). Total light emission consists of intrinsic radiative recombination and exciton-SP coupling. Therefore, given *n*_*i*_(*t*), the total PL intensity is calculated using Equation (4) assuming all of the surface plasmons generated from excitons relax to emit light and no re-absoption of PL by QDs.

Exciton-SP coupling can be considered as Förster resonance energy transfer as discussed earlier and assumed to follow the same distance dependence. The coupling rate(*k*_*SP*_) as a function of distance is defined as follows.


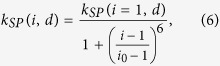



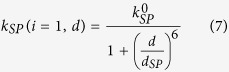


*k*_*SP*_(*i* = 1, *d*) is the rate constant for the coupling between the first bottom QD monolayer on Si_3_N_4_ films (of thickness *d*) and silver SP modes. *d*_*SP*_ is the characteristic distance that yields half the maximum constant 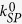 when QDs are directly on the silver film. *i*_0_ is the characteristic number which indicates the coupling efficiency becomes the half of the maximum value *k*_*SP*_(*i* = 1, *d*) in the QDs medium.

Charge transfer or tunneling must be also taken into account because it decays exponentially with increasing Si_3_N_4_ film thickness. The rate constant for electron tunneling can be described as an exponential decay function as below.


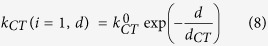



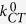
 is the rate constant when QDs are directly on the silver film. *d*_*CT*_ is the tunneling distance. We assume that electron tunneling takes place only between the first monolayer and the silver metal. For excitons generated in the upper layers initially, they are assumed to move downward first through energy transfer between adjacent QDs and then to be dissociated by electron tunneling once they reach the first QD monolayer. This means that *k*_*CT*_ (*i* ≥ 2, *d*) = 0 in Equation (1) and (3).

The variation of the total PL intensity from QD monolayers as a function of Si_3_N_4_ thickness (the inset of Fig. 2(a)) is written simply by


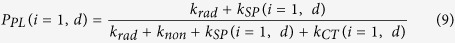


Using Equation (7)–(9), 13, 15, a fitting analysis for the distance-dependent PL intensity of Fig. 2(b) yields the fitting curves with the kinetic parameters. The results indicate that 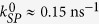, *d*_*SP*_ ≈ 36 nm 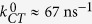, and *d*_*CT*_ ≈ 1.5−3.0 nm.

Instead of solving the coupled Equation (1)–(3), 13, 10, an insightful and straightforward scheme can simulate the total PL intensity from multilayer QD films on the silver resonator in Fig. 5 using Equation (10) as follows.


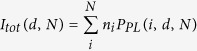



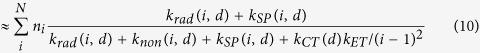


Equation (10) assumes that the transient exciton density at the *i*^*th*^ layer (*n*_*i*_) is decoupled from the density of adjacent layers, *n*_*i*−1_and *n*_*i*+1_ in Equations (1)–(3)., 13, 10 Instead, the excitons generated at the *i*^*th*^ layer are assumed to move directly towards the QD/silver interface at the rate of *k*_*ET*_/(*i*−1)^2^and undergo exciton dissociation by charge transfer or tunneling. The term of *k*_*ET*_/(*i*−1)^2^ in the denominator originates from the fact the diffusion time is proportional to the square of the diffusion length. *k*_*rad*_(*I*,*d*) is assumed to be constant as 0.005 ns^−1^ regardless of *i* and *d*. Using single- and multilayer PL data for *d* = 100 nm in Fig. 5, *k*_*non*_(*i* = 1, *d*) an*d k*_*non*_(*i* ≥ 2, *d*) were estimated 0.29 ns^−1^ and 0.071 ns^−1^, respectively. The value at the interface is about four times larger than *k*_*non*_(*i* ≥ 2, *d*) *d*ue to charge transfer to the Si_3_N_4_ film as shown in Fig. 1(b). The distance-dependent rate constant for exciton-SP coupling, *k*_*SP*_(*i*,*d*) can be obtained using Equation (6). Using the parameters obtained previously and Equation (5, 6, 7, 8, 9, 10)the total PL intensities (represented by solid shapes) from QD multilayers were fitted as shown by open shapes in Fig. 5. The experimental data are in good agreement with the fitting curves. The tunneling distance *d*_*CT*_ of 1.5 nm was used. The characteristic number, *i*_0_ *i*n Equation (6) was estimated as 6 for *d* = 0 nm, 5 for *d* = 10 nm, and 3 for *d* = 20 nm, respectively. This indicates that the Si_3_N_4_ film thickness of ~10 nm shows the effects similar to one or two QD layers in the distance dependence of exciton-SP coupling as discussed earlier. The rate constant for energy transfer, *k*_*ET*_ was estimated ~0.040 ns^−1^. The dimensionless skin depth was yielded as ~10 for the best fitting of the enhancement at *d* = 0, 10, and 20 nm, but it seems underestimated because the intensity deviates appreciably with increasing QD layers for *d* = 100 nm. More precise fitting parameters and smaller deviations may be obtained by considering re-absorption of PL by other QDs, exciton diffusion, and back reflection from the silver metal.

## Additional Information

**How to cite this article**: Shin, T. *et al*. Exciton Recombination, Energy-, and Charge Transfer in Single- and Multilayer Quantum-Dot Films on Silver Plasmonic Resonators. *Sci. Rep*. **6**, 26204; doi: 10.1038/srep26204 (2016).

## Supplementary Material

Supplementary Information
