# Supplementary material for: Exciton Recombination, Energy-, and Charge Transfer in Single- and Multilayer Quantum-Dot Films on Silver Plasmonic Resonators
Source: Sci Rep. 2016 May 17;6:26204. doi: 10.1038/srep26204 (PMC4869000; doi:10.1038/srep26204)
Supplement: Supplementary Information [file srep26204-s1.pdf]

Supplementary Information

Exciton Recombination, Energy-, and Charge Transfer in Single- and Multilayer Quantum-Dot Films on Silver Plasmonic Resonators

Taeho Shin[1,5], Kyung-Sang Cho[2], Dong-Jin Yun[1], Jinwoo Kim[3], Xiang-Shu Li[1], Eui-Seong Moon[1],Chan-Wook Baik[2], Sun Il Kim[2], Miyoung Kim[4], Jun Hee Choi[2],[*] Gyeong-Su Park[1], Jai-Kwang Shin[1], Sungwoo Hwang[2], and Tae-Sung Jung[2]

[1]Analytical Science Group, Samsung Advanced Institute of Technology, Suwon 443-803, Republic of Korea
[2]Device & System Research Center, Samsung Advanced Institute of Technology, Suwon 443-803, Republic of Korea
[*]joonie.choi@samsung.com
[3]Department of Materials Science and Engineering, Frederick Seitz Materials Research Laboratory, University of Illinois at Urbana-Champaign, Urbana, IL 61801, USA
[4]Department of Materials Science and Engineering, Seoul National University, Seoul 151–744, Republic of Korea
[5]Department of Chemistry, Chonbuk National University, Jeonju 561-756, Republic of Korea

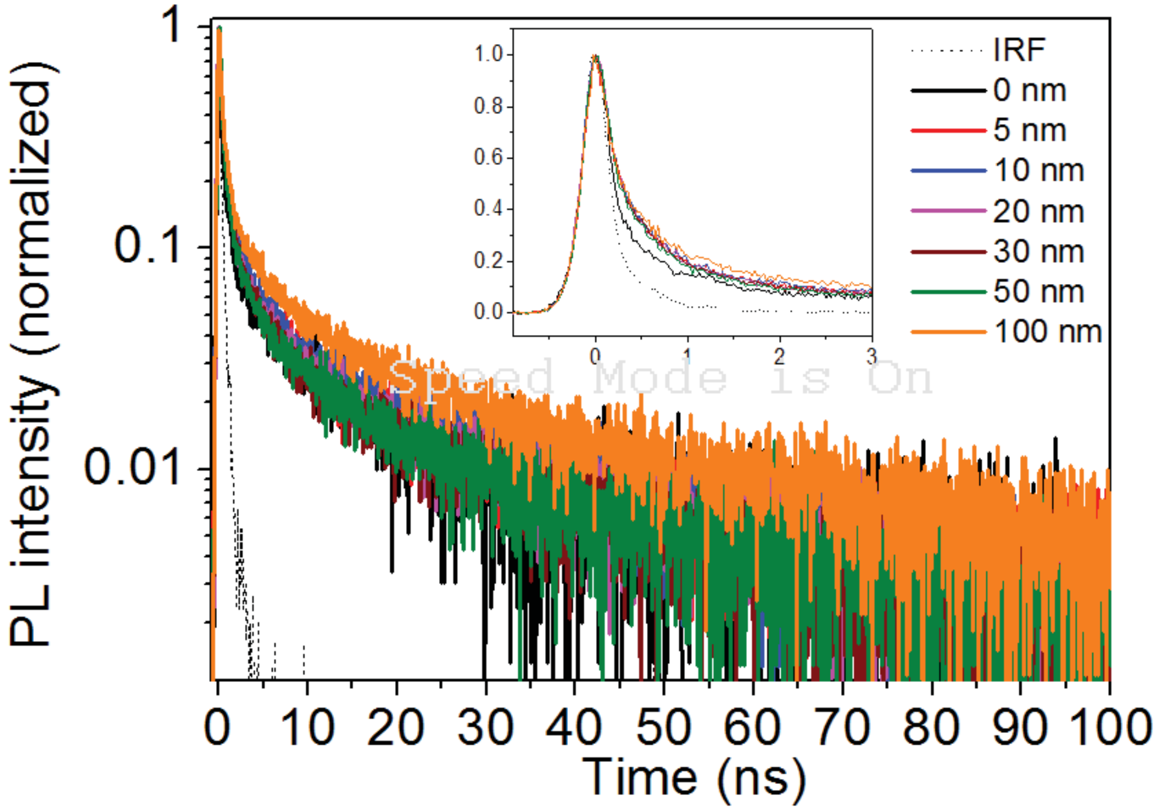

Figure S1. Log plot of the PL decay curves in Figure 1(d).

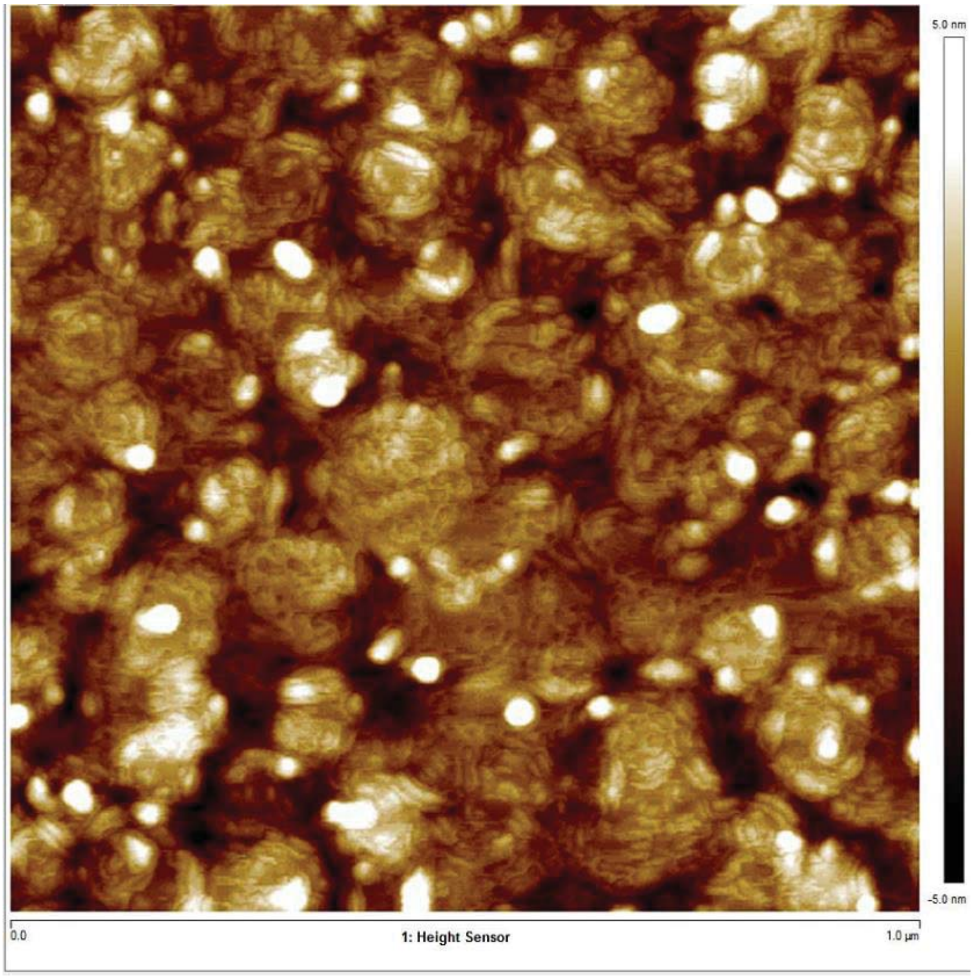

Figure S2. AFM image of silver films.

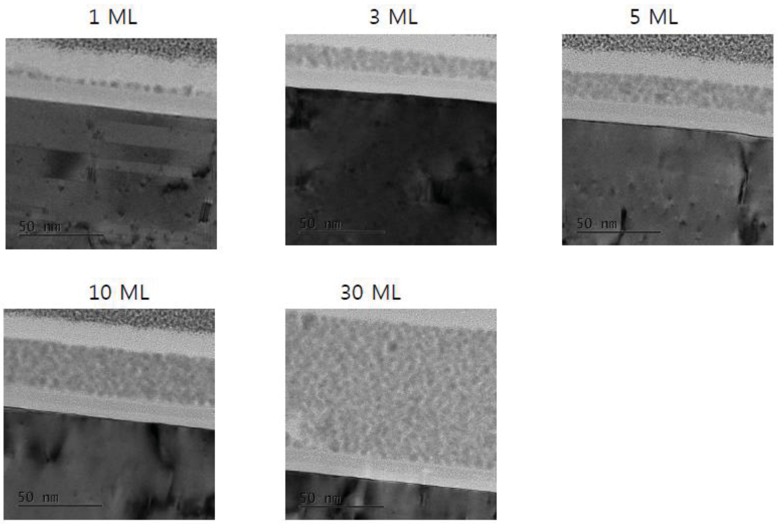

Figure S3. TEM images of QD films with different thicknesses.

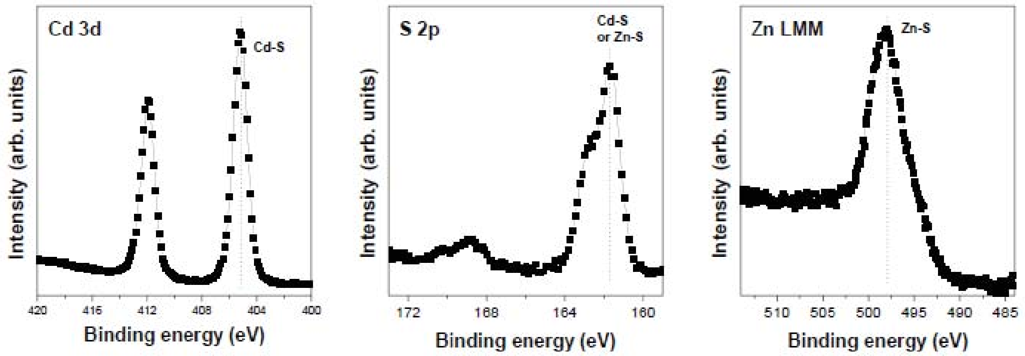

Figure S4. XPS spectra from QD films.

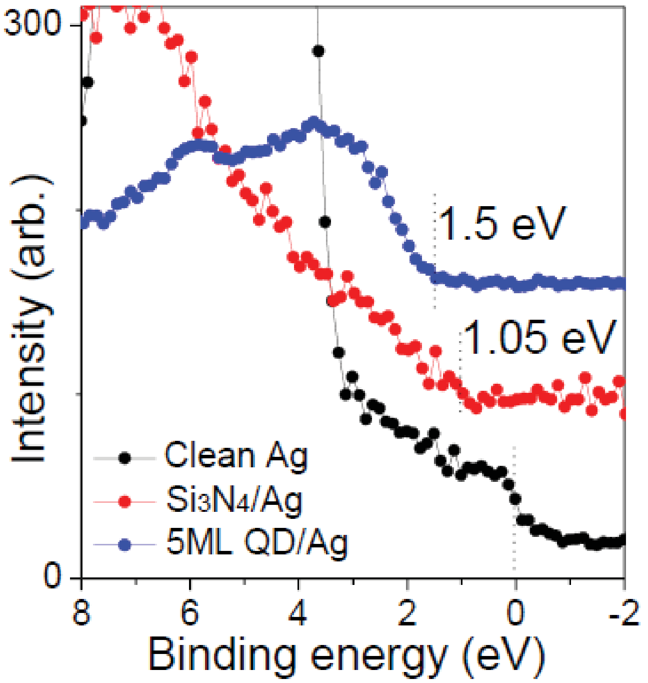

Figure S5. XPS spectra from QDs, Si_3N_4, and Ag.
